# Evaluating treatments in rare indications warrants a Bayesian approach

**DOI:** 10.3389/fphar.2023.1249611

**Published:** 2023-09-20

**Authors:** Emma K. Mackay, Aaron Springford

**Affiliations:** Cytel, Toronto, ON, Canada

**Keywords:** Bayesian, statistical methods, health economics and outcomes research (HEOR), evidence synthesis, real-world evidence (RWE), rare diseases, comparative effectiveness

## Abstract

Evaluating efficacy and real-world effectiveness for novel therapies targeting rare mutations or patient subpopulations with unmet needs is a growing challenge in health economics and outcomes research (HEOR). In these settings it may be difficult to recruit enough patients to run adequately powered randomized clinical trials, resulting in greater reliance on single-arm trials or basket trial designs. Additionally, evidence networks for performing network meta-analysis may be sparse or disconnected when comparing available treatments in narrower patient populations. These challenges create an increased need for use of appropriate methods for handling small sample sizes, structural modelling assumptions and more nuanced decision rules to arrive at “best-available evidence” on comparative and non-comparative efficacy/effectiveness. We advocate for greater use of Bayesian methods to address these challenges as they can facilitate efficient and transparent borrowing of information across varied data sources under flexible modelling assumptions, probabilistic sensitivity analysis to assess model assumptions, and more nuanced decision-making where limited power reduces the utility of classical frequentist hypothesis testing. We illustrate how Bayesian methods have been recently used to overcome several challenges of rare indications in HEOR, including approaches to borrowing information from external data sources, evaluation of efficacy in basket trials, and incorporating non-randomized studies into network meta-analysis. Lastly, we provide several recommendations for HEOR practitioners on appropriate use of Bayesian methods to address challenges in the rare disease setting.

## Introduction

A core task of health economics and outcomes research (HEOR) is to compare the effectiveness of two or more competing treatments. Over the past several decades, researchers in HEOR have been working to realize the promise of a “big data” revolution in which an excess of evidence can be brought to bear on any given decision problem (Berger and Doban, 2014). However, due to advances in health technologies which target smaller populations and/or very rare diseases we continue to see challenges of limited data and small sample sizes. In response to these trends, we advocate for modern Bayesian approaches which can incorporate all available information in a principled and transparent way. In our view, Bayesian approaches are particularly valuable if primary data sources are insufficient to establish reliable and statistically conclusive superiority of a novel treatment compared to the *status quo*. In these cases, the novel treatments that are urgently needed by patients may be passed over if typical large-sample, dichotomous statistical significance thresholds are treated as an unquestioned default by decision-makers.

While Bayesian methods have seen substantial uptake in the area of meta-analysis–for example, in guidance from the UK National Institute for Health and Care Excellence’s (NICE) Decision Support Unit (DSU) (Dias et al., 2011)–, we suggest that significant gains can also be made in rare disease settings where sample sizes and available evidence bases are more limited. A goal of this paper is to provide examples and guidance on how practitioners can incorporate external information using Bayesian modelling to address some of the challenges of evaluating efficacy/effectiveness that arise in health technology assessments (HTA) of newly developed therapies for rare indications. We point to some key applications in which we believe important gains can be made: borrowing from external sources to augment a concurrent control arm or to estimate a historical control rate for rare diseases; incorporating disparate data sources (such as randomized controlled trial (RCT) and non-randomized study (NRS) data) into a meta-analysis; and applying Bayesian hierarchical models (BHM) to partially pool information across heterogeneous data sources. In each of these applications, common questions emerge: 1) What relevant information can we draw on to improve existing analyses and estimates? 2) When existing data are limited, what assumptions might enable incorporation of external information, and are these plausible? Or, when very strong assumptions are needed, what would constitute “best-available evidence”? And 3) how can we characterize the limitations of the analysis and assess sensitivity to violations of key assumptions?

### What are Bayesian methods and why use them?

Bayesian inference defines a probability model for data which is a function of parameters (the likelihood), and a probability model for parameters before any data are observed (the prior). After data are observed, the prior and the likelihood are used to calculate a probability distribution for the parameters given the data (the posterior). If there is information available which is related directly to the model parameters, it can be included in the prior. If there is information available in the form of additional data from another source, it can be included in the likelihood. The posterior distribution contains all available information about the model parameters, and in practice is a very useful mathematical object. For example, functions of the posterior such as the probability that one treatment is superior to another, or the expected benefit of selecting one treatment over another, or the distribution of predicted patient outcomes in a given population, can all be obtained without much additional computational effort. In the frequentist approach, many of these derived quantities are not available, and even if they are available their calculation is considerably more burdensome.

Because Bayesian methods lead to probability statements about model parameters, they are vital to formal decision analysis and thus HTA (see Spiegelhalter et al. (1999) and examples therein). Bayesian inference leads to statements like: “there is a 95% probability that the hazard ratio is between 0.6 and 0.84”; whereas frequentist inference leads to statements such as: “if the trial were repeated many times, and a 95% confidence interval constructed for each, the true hazard ratio would be within 95% of the intervals.” In an HTA context, we argue that the former is not only more interpretable, but also more directly useful for decision making. A more extensive comparison of Bayesian and frequentist methods can be found in Spiegelhalter et al. (1999).

### How can Bayesian borrowing help bolster limited sample sizes in HEOR analyses?

Bayesian borrowing methods can incorporate information about model parameters (e.g., the control arm response rate) from external data in a transparent manner. These methods allow for down-weighting of the external data to mitigate potential bias arising from different parameter values in the current population compared to the external populations. One established approach is to borrow information by means of a power prior (Ibrahim and Chen, 2000; Ibrahim et al., 2015). The power prior is formed by taking a prior for the parameter and combining it with a discounted likelihood for the parameter on the external data. The external data parameter likelihood is discounted by raising it to the power of a discount parameter between 0 and 1. A discount parameter value of 0 corresponds to no borrowing and a value of 1 yields complete pooling of the datasets. Due to the challenge of selecting a value for the discount parameter, one option for practitioners is to vary the discount parameter and assess how much borrowing from the external data is required before a specified decision threshold—or “tipping point”—is reached (e.g., for concluding that a treatment is effective). This sort of tipping point analysis has precedent in a regulatory context when using Bayesian borrowing (US Food and Drug Administration, 2018). Another option is to use dynamic borrowing, in which the discount parameter is treated as a random quantity with its own prior distribution. In theory, this approach allows the amount of borrowing to depend on the degree of agreement in observed outcomes between the current and external data sources. In practice, there may not be much information available in the data about the discount parameter, and results can be sensitive to the choice of prior. Regardless, proper implementation of dynamic borrowing using a power prior requires a normalization step (Neuenschwander et al., 2009) which can be computationally challenging to implement. Ibrahim et al. (2015) provide a more detailed overview of power priors, including extensions such as commensurate priors, for interested readers. Additionally, Viele et al. (2014) compare several approaches to borrowing from historical data, including the use of power priors.

Another prior-based approach to Bayesian borrowing is to formulate a meta-analytic predictive (MAP) prior for the parameter of interest (Neuenschwander et al., 2010). As an example, suppose we want to borrow information on the response rate for the control treatment. We conduct a Bayesian meta-analysis (typically a random effects meta-analysis) to obtain the posterior predictive distribution for the control treatment response rate. This posterior then becomes the prior for the response rate in our concurrent control arm—if there is one—or represents the entirety of information available for this parameter if there is no concurrent control arm. The posterior predictive distribution is preferred because it incorporates heterogeneity in response rates across trial populations, and we seek to generalize from the external populations to our current population. A narrower/more precise MAP prior in effect represents a larger sample size being borrowed from the external data. In cases where the generalization from external to current is insufficiently conservative, robust MAP priors have been used (Schmidli et al., 2014). Robust MAP priors are defined as a weighted mixture of the MAP prior and a vague prior. This approach is analogous to the power prior in that placing more weight on the vague component in the mixture results in a more diffuse prior distribution which imparts less information, down-weighting the contribution of the external data.

Power prior and robust MAP prior methods have different strengths and weaknesses in practice. Power prior methods can be more challenging to implement (especially when the discount parameter is a random quantity), but they have a simple form and can easily be adapted to incorporate disparate sources of external information. MAP prior methods will be more familiar to those experienced with Bayesian meta-analysis, and may be easier to explain and justify in many HEOR settings. Both approaches can incorporate aggregate data and/or individual patient data (IPD) from multiple sources, and both can be used for tipping point analysis if desired (US Food and Drug Administration, 2018; Best et al., 2021). In one prospective RCT using robust MAP to reduce control group allocation, variance of the robust MAP prior was inflated to achieve a target effective sample size (Richeldi et al., 2022)—a practical approach to borrowing which could also be applied to a power prior with fixed discount parameter.

### How can we model structural relationships between data sources while also accounting for potential heterogeneity?

In cases where a structural relationship among data sources can be assumed, Bayesian hierarchical models (BHMs) are another option for partial pooling of information in which hierarchical dependencies of key parameters are modeled explicitly (Gelman et al., 2013). BHMs assume that some model parameters are related by virtue of being drawn from a common distribution—i.e., that they are exchangeable—but that the parameters of the distribution are themselves random quantities. For example, response rates for a specific control treatment are often assumed to be heterogeneous across data sources but nonetheless may be interrelated. Under a BHM approach, information on the control treatment response rate can be partially pooled across data sources, with the amount of pooling dependent on the degree of heterogeneity in response rates across data sources (less borrowing occurs if response rates are very heterogeneous). This also has the effect of shrinking parameter estimates towards the grand mean, mitigating overfitting and improving inference for individual parameters, particularly when data are limited (Gelman et al., 2013).

To illustrate the utility of BHMs in the HEOR space, we focus on some recent applications to analyses of basket trials. Basket trial designs include patients with multiple cancer types which share a common targetable mutation or biomarker. In these basket trials, sample sizes tend to be extremely limited, treatment responses are expected to vary among tumour types, and control arms are often omitted. Murphy et al. (2021) use a BHM approach in a single-arm basket trial setting for evaluating response for NTRK fusion-positive patients receiving larotrectinib. Their approach allows for partial borrowing of information on response rates across tumour types to produce estimates of response for individual tumour types, the overall basket of represented tumours, and for an unrepresented histology. BHM approaches were also well-received in a NICE technical appraisal for larotrectinib (UK National Institute for Health and Care Excellence, 2020).

In the BHM approach to analysis of basket trials, exchangeability of tumour types may be a clinically tenuous assumption (although perhaps an acceptable approximation in light of data limitations if the BHM is flexible enough to describe the data). Neuenschwander et al. (2016) propose an exchangeable-non-exchangeable (EXNEX) model which allows for relaxation of strong exchangeability assumptions, and we envision future methodological developments in this area. Mackay et al. (Mackay et al., 2022; Mackay et al., 2023) have recently proposed an extension of BHM modelling for histology-independent therapies to allow for indirect treatment comparisons (ITC) between multiple basket trials. The approach allows for adjustment for potential confounding due to differences in tumour type compositions between trials while preserving limited precision/power by means of partial pooling. The reader is directed to Murphy et al. (2022) for a more detailed discussion of modelling approaches for histology-independent therapies in an HTA context.

While hierarchical models can be implemented under both a Bayesian and classical frequentist approach, a key advantage of Bayesian approaches is the ability to incorporate prior information and perform probabilistic sensitivity analyses when faced with challenging settings with limited available data. For example, use of weakly informative priors can avoid issues of extreme overfitting to the data. Additionally, it can be difficult to reliably estimate the heterogeneity parameters for a hierarchical model when the number of groups (e.g., tumour types) is very small. In these situations, multiple prior distributions can be used to assess how sensitive conclusions are to assumptions about the degree of heterogeneity in outcomes across groups.

Beyond applications to basket trials, BHMs have been used to incorporate disparate data sources, structural assumptions, and borrowing approaches when no single source is sufficient for inference and decision-making. For example, Heeg et al. (2022) recently used BHMs to partially pool information on specific model parameters across a class of immune-oncology therapies to improve survival extrapolations from immature data.

### Can we incorporate non-randomized studies into meta-analyses while mitigating risk of bias to address challenges in assessing comparative efficacy/effectiveness?

Meta-analyses which synthesize the published evidence on relative treatment effects generally rely on RCT evidence only. However, when estimating real world effectiveness or efficacy/effectiveness in key patient populations of interest, or when RCT evidence is lacking due to the rarity of some indications, incorporating information from non-randomized studies (NRS) using real-world data becomes appropriate (Sarri et al., 2022). Relevant NRS would include cohort studies comparing patient outcomes by treatment using appropriate methods to mitigate sources of bias (Faria et al., 2015)—particularly well-designed synthetic control arm analyses (Thorlund et al., 2020) and target trial emulations (Hernán and Robins, 2016). Sarri et al. (2022) provide a structured framework for incorporating NRS into meta-analyses—a process which includes assessing risk of bias in the identified NRS and careful selection of methods to appropriately down-weight the influence of NRS, to incorporate bias adjustments, and to conduct sensitivity analyses to the modelling decisions.

Several promising approaches exist for incorporating NRS into a network meta-analysis (NMA) or pairwise meta-analysis which are both conducive to down-weighting the NRS either statically or dynamically, and to probabilistic sensitivity analysis. Schmitz et al. (2013) highlight three approaches to incorporating NRS: 1) naïve pooling of the RCT and NRS evidence, 2) incorporation of the NRS using informative priors, and 3) use of a hierarchical model to capture the potential heterogeneity in relative treatment effects between RCT and NRS. They also outline how corrections for systematic and non-systematic bias can be incorporated into approaches 2) and 3).

Schmitz et al. (2013) note that the bias in NRS relative treatment effects (e.g., log-odds ratios, log-hazard ratios, *etc.*) can be modelled using a Gaussian distribution where the mean and variance represent systematic and non-systematic components of the bias, respectively. This allows for incorporation of NRS data into the meta-analysis with potential bias adjustment and down-weighting—either by means of a bias-adjusted priors or through direct incorporation into the likelihood. Efthimiou et al. (2017) highlight additional approaches than can be taken to form priors from NRS data—such as down-weighting of the parameter likelihood from the NRS data by means of a power prior or mixture prior (e.g., robust MAP priors). Verde et al. propose a hierarchical meta-regression (HMR) approach which can be used to estimate a bias-correction term for study design or other study-level covariates, and detect and down-weight outlier studies when there is significant cross-study heterogeneity (Verde et al., 2016; Verde, 2017; Verde, 2019). Additionally, HMRs can be used to extrapolate treatment effects to specific populations when IPD is available for at least one study or real-world data source.

## Discussion

As new drug development is focusing more and more on narrower indications, HEOR practitioners are increasingly faced with challenges of limited data. These limitations arise from difficulties recruiting enough patients to conduct adequately powered RCTs (leading to more reliance on single-arm trials for regulatory and HTA submissions), narrowing of indications or subpopulations of interest leading to smaller numbers of relevant studies being identified in systematic literature reviews (and greater risk of disconnected or tenuous networks in NMAs), and more reliance on evidence from ITCs that are unlikely to yield precise estimates of relative treatment effects. Consequently, we present several recommendations for how Bayesian methods (including many of the approaches outlined above) can be used to help mitigate some of these pitfalls.

Firstly, since Bayesian approaches allow for weakly informative priors to be specified before analyzing the data, use of sensible default priors can mitigate some of the risks of model overfitting when data are very limited without imposing overly strong assumptions. With weakly informative defaults, the prior can be easily overwhelmed when informative data are available. An example of this can be found in the Keefe et al. (2021) meta-analysis of diagnostic tests where use of weakly informative priors allows for the meta-analysis to be run even when the model is overparametrized for some classes of diagnostic tests (too few studies relative to the number of parameters). In these cases, the prior is minimally updated (or not updated at all) and continues to reflect agnostic beliefs as to whether the test is predictive. In cases where more studies are available, the prior is updated to reflect the larger evidence base.

Secondly, if strong modelling assumptions are needed to synthesize the limited amount of available data, probabilistic sensitivity analyses should be conducted to assess robustness to deviations from these assumptions. For example, if it is infeasible to conduct a random effects NMA due to too few studies in the network, different heterogeneity assumptions can be assessed by fitting modified random effects NMAs in which different strong priors are used for the heterogeneity parameters, each reflecting a plausible scenario. In this context, fixed effects NMA can be viewed as a special case of random effects NMA, and use of informative priors on heterogeneity parameters allows for sensitivity analysis even when data are too limited to estimate these parameters.

Lastly, if precision/power are anticipated to be extremely limited (e.g., in a rare disease setting), it may be worth considering a context-appropriate decision rule rather than a default *p*-value threshold. For example, if we are performing an ITC between two treatments that have received regulatory approval based on single-arm trials, and it is infeasible to conduct an adequately powered ITC, it may be sensible to prioritize reimbursement of one drug over the other based on the posterior probability of superiority (a quantity which is directly available in Bayesian inference). This would arguably constitute a “best-available evidence” standard in this example.

In summary, Bayesian methods provide a principled framework for quantifying the amount of evidence in favour of a particular conclusion, are well-suited to combining information from multiple data sources under various structural assumptions, and can facilitate probabilistic sensitivity analyses to probe these structural assumptions. For these reasons we believe that Bayesian methods should play an increasing role in health economics and outcomes research.

## Data Availability

The original contributions presented in the study are included in the article/Supplementary Material, further inquiries can be directed to the corresponding author.
